# The Interplay of Splicing and Metabolism in Cancer

**DOI:** 10.3390/cells15121117

**Published:** 2026-06-20

**Authors:** Dillon M. Voss, Yange Cui, Peter S. Klein

**Affiliations:** 1 Renaissance School of Medicine, Stony Brook University, Stony Brook, NY 11794, USA; 2 Division of Hematology-Oncology, Department of Medicine, Perelman School of Medicine, University of Pennsylvania, Philadelphia, PA 19104, USA

**Keywords:** splicing, metabolism, retrograde signaling, cancer, leukemia, myelodysplasia, mitophagy, pyruvate kinase, spliceosome, PINK1

## Abstract

Aberrant RNA splicing and metabolic reprogramming are defining hallmarks of cancer that were historically studied as parallel processes. Increasing evidence now reveals extensive crosstalk between these pathways, whereby RNA splicing reshapes metabolic circuits, and metabolic states reciprocally influence splice-site selection and spliceosome activity. In this review, we synthesize recent mechanistic insights into how splicing programs regulate metabolic adaptation across diverse cancer contexts. We discuss recurrent oncogenic mutations in spliceosomal components and dysregulation of RNA-binding proteins (RBPs) that drive alternative splicing events in key metabolic regulators, which promote metabolic plasticity required for tumor growth. We further examine how metabolites and nutrient-sensing pathways directly modulate splicing factor activity, spliceosome dynamics, and RNA processing. We also summarize a new mechanism of mitochondrial quality control mediated by retrograde signals from mitochondria to the spliceosome to enhance mitophagy of dysfunctional mitochondria.

## 1. Introduction

Cancer cells continuously adapt under fluctuating environmental conditions, including changes in nutrient availability, oxygen levels, and cellular stress [1]. Two biological processes that enable rapid and reversible adaptation are RNA splicing and cellular metabolism [2,3]. Regulated splicing can rapidly reshape gene expression by altering transcript isoform expression without changes in DNA sequence or transcription, while metabolic networks dynamically adjust nutrient utilization and biosynthetic flux to sustain proliferation [4,5,6]. As both processes operate on relatively short timescales and exert broad influence over cellular physiology, they provide powerful mechanisms through which cancer cells can rapidly reprogram their functional state to survive [3].

Consistent with this idea, dysregulation of splicing and metabolism is widely observed across tumor types [1,2,7]. Somatic mutations in spliceosomal components as well as altered expression of splicing factors can drive oncogenesis. Oncogenic signaling pathways that modulate spliceosome activity can also drive widespread changes in splicing programs that may contribute to malignancy [8]. In parallel, cancer cells undergo metabolic reprogramming to support rapid growth and survival, reshaping pathways such as glycolysis, glutamine metabolism, and one-carbon metabolism to generate energy and biosynthetic precursors [4,5,9].

While splicing and cellular metabolism have traditionally been studied in separate contexts, growing evidence indicates that they are frequently interconnected. Alternative splicing can directly regulate metabolic pathways by generating enzymatic isoforms with distinct catalytic properties or regulatory functions. Conversely, metabolic states and metabolite availability can influence RNA processing by modulating spliceosome activity, splicing factor modifications, and chromatin environments that shape splice-site selection. The role of altered splicing in cancer has been extensively and authoritatively reviewed elsewhere [2,7,8,10,11,12,13,14]; metabolic changes associated with malignancy [4,5,9] and the metabolic regulation of splicing have also been independently reviewed in detail [3,15], but reviews on the interaction of splicing and metabolism in cancer are more limited. In this review, we examine recent insights into splicing and metabolism and discuss how their reciprocal regulation contributes to cancer pathogenesis and adaptation.

## 2. Regulation of Pre-mRNA Splicing

Splicing is the process by which introns are removed from newly transcribed precursor messenger RNA (pre-mRNA) and exons are joined to produce a mature mRNA transcript containing the protein coding sequence and 5′ and 3′ untranslated regions (UTRs) [16,17]. Splicing is carried out by the spliceosome, a ribonucleoprotein complex [18] with a catalytic core of the five small nuclear ribonucleoproteins (snRNPs) U1, U2, U4, U5, and U6. snRNPs assemble on conserved pre-mRNA splice-site elements and catalyze intron removal [18]. These elements include the 5′ splice site located at the 5′ end of the intron, the branch point sequence and polypyrimidine tract within the intron, and the 3′ splice site located at the intron–exon boundary at the 3′ end of the intron [18]. The spliceosome mediates two transesterification reactions that excise the intron and ligate adjacent exons [18].

Auxiliary RNA-binding proteins (RBPs) and regulatory factors assist in defining splice sites and modulating spliceosome assembly [19]. The U2 auxiliary factor (U2AF) complex aids in recruiting the U2 snRNP to the 3′ splice site [20]. The large subunit U2AF2 (U2AF65) binds the polypyrimidine tract (PPT) located upstream of the 3′ splice-site, while the smaller subunit U2AF1 (U2AF35) recognizes the conserved “AG” dinucleotide sequence at the 3′ splice site [21]. These interactions promote recruitment of the U2 snRNP to an upstream adenosine known as the branch point [21]. Additionally, the SF3b complex stabilizes branch point recognition by the U2 snRNP and helps position the branch point adenosine for splicing [22,23].

In addition, RBPs bind regulatory sequences within exons or introns to influence spliceosome recruitment [24]. For example, members of the serine/arginine-rich splicing factor (SRSF) family bind exonic splicing enhancer (ESE) elements and promote spliceosome assembly by enhancing recognition of both splice sites, in part through facilitating recruitment and stabilization of U1 snRNP at the 5′ splice site and the U2AF complex at the 3′ splice site, thereby strengthening exon definition [25,26,27,28]. Conversely, heterogeneous nuclear ribonucleoproteins (hnRNPs) bind exonic and intronic splicing silencer (ESS and ISS) elements and inhibit spliceosome assembly at nearby splice sites by occluding splice sites or disrupting recognition by U1 snRNP and U2AF [24,29,30].

Disruption of these regulatory proteins is frequently observed in cancer [8,14,31]. Recurrent somatic mutations in spliceosomal components, including SF3B1, SRSF2, U2AF1, and ZRSR2, account for >60% of driver mutations in hematologic malignancies such as myelodysplastic syndromes (MDS), chronic myelomonocytic leukemia (CMML, an MDS/myeloproliferative overlap neoplasm), chronic lymphocytic leukemia (CLL), and acute myeloid leukemia (AML), where they drive widespread alterations in splicing [14,32,33,34,35,36]. These mutations often alter splice-site recognition and promote aberrant splicing events such as cryptic splice-site usage, exon skipping, and intron retention [36]. For example, *SRSF2^P95H/+^* mutations promote retention of a poison exon in EZH2, leading to reduced expression of EZH2 in MDS and AML [37,38,39,40]. In addition to mutations, altered expression of regulatory factors such as SRSF1 can promote oncogenic splicing programs that support proliferation, survival, and metabolic adaptation in cancer cells [41,42].

Splice-site selection is also influenced by co-transcriptional mechanisms linked to RNA polymerase II (Pol II) kinetics and chromatin state [43]. The rate of Pol II elongation can modulate exon recognition, where slower elongation provides an extended temporal window for spliceosome assembly at weak splice sites, which can promote either exon inclusion or skipping depending on the regulatory context [44]. Chromatin structure and histone modifications further contribute to this process by regulating Pol II kinetics and by recruiting splicing factors through adaptor proteins that couple transcription to RNA processing [45,46]. These findings support a model in which splice-site selection is governed not only by local RNA sequence elements and RBPs, but also by the broader transcriptional and epigenetic context.

## 3. Alternative Splicing Increases Transcriptome Diversity

More than 90% of the genes in higher eukaryotes undergo alternative splicing [47], generating multiple mRNA isoforms that encode proteins with distinct structural and/or regulatory properties and allow cells to dynamically adjust gene expression programs in response to developmental cues, environmental signals, and cellular stress. These isoform differences can have profound functional consequences. For example, alternative splicing of BCL2L1 generates either the anti-apoptotic isoform BCL-xL or the pro-apoptotic isoform BCL-xS, thereby directly influencing cell survival decisions [48].

Common modes of alternative splicing include exon skipping (cassette exons), mutually exclusive exon usage, alternative 5′ splice-site selection, alternative 3′ splice-site selection, and intron retention [49]. Among these, exon skipping is the most prevalent form in mammals [47]. Mutually exclusive exon usage ensures that only one of two neighboring exons is incorporated into the final transcript, whereas alternative 5′ or 3′ splice-site selection involves the use of distinct donor or acceptor splice sites within the same exon. Intron retention can introduce new coding sequences and may contain in-frame premature termination codons (PTCs) that can lead to nonsense-mediated decay. Collectively, these different splicing patterns expand transcriptome diversity and provide a versatile mechanism for regulating gene expression and protein function.

Alternative splicing decisions are highly responsive to cellular signaling pathways and post-translational modifications that regulate the activity of splicing factors [24]. Many splicing regulators, including SR proteins and hnRNPs, are controlled through post-translational modifications. For example, phosphorylation by protein kinases including CMGC family kinases SR protein kinases (SRPKs), CDC-like kinases (CLKs), dual-specificity tyrosine-phosphorylation-regulated kinases (DYRKs), and glycogen synthase kinase-3 (GSK-3a/b), influences splicing factor localization, RNA-binding activity, and ability to recruit spliceosomal components [50,51,52,53,54,55,56]. Additional post-translational modifications, including arginine methylation, acetylation, ubiquitination, and O-GlcNAcylation, can further influence spliceosome dynamics and splice-site selection [57,58]. Moreover, RNA modifications such as N6-methyladenosine (m6A) can also affect splicing by altering RNA structure or recruiting RNA-binding proteins that modulate spliceosome recruitment [59]. Through these mechanisms, alternative splicing can rapidly respond to changes in cellular signaling, metabolic state, and environmental stress.

## 4. Metabolic Reprogramming in Cancer

Cancer cells exhibit multiple layers of metabolic rewiring that both alter splicing and are modulated by alternative splicing. Over a century ago, Otto Warburg observed that tumor tissues preferentially take up glucose and convert it to lactate in the presence of oxygen, a phenomenon known as aerobic glycolysis, or the Warburg effect [1,9,60]. Aerobic glycolysis supports anabolic growth by diverting glycolytic intermediates into biosynthetic pathways [61,62]. In addition, glutamine metabolism provides a major source of carbon and nitrogen for anaplerosis and biosynthesis, while one-carbon metabolism integrates inputs from serine and glycine to support nucleotide production and methylation reactions [63,64]. Lipid biosynthesis is frequently upregulated to support membrane formation and signaling, and alterations in nitrogen handling pathways, including the urea cycle, further enable sustained anabolic growth [65,66]. These metabolic programs respond to environmental conditions such as nutrient availability, hypoxia, and oncogenic signaling pathways including MYC, mutant p53, and PI3K-AKT-mTOR [61]. In the following sections, we highlight how alternative splicing contributes to this metabolic reprogramming by generating distinct metabolic enzyme isoforms.

## 5. Enzymatic Isoforms That Influence Cancer Metabolism

### 5.1. Pyruvate Kinase

**Figure 1:**
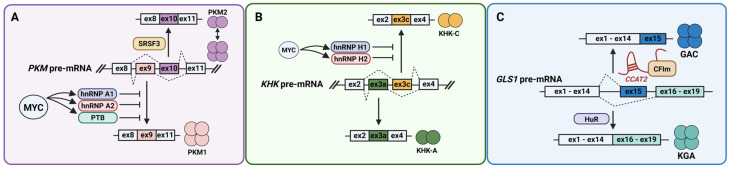
Alternative splicing reprograms metabolic enzyme function in cancer. (**A**). *PKM* mutually exclusive alternative splicing of exon 9 vs. exon 10. PKM1 is a constitutively active tetramer that promotes pyruvate flux, oxidative phosphorylation, and ATP production in differentiated tissues. PKM2, preferentially expressed in cancer, exhibits lower activity and supports diversion of glycolytic intermediates into anabolic pathways. This splicing decision is regulated by hnRNPA1, hnRNPA2, and PTB, which repress exon 9, and SRSF3, which promotes exon 10 inclusion downstream of oncogenic signaling. (**B**). *KHK* mutually exclusive alternative splicing of exon 3a vs. exon 3c. KHK-C exhibits high kinase activity and promotes fructose-driven glycolytic and lipogenic flux, whereas KHK-A has lower catalytic activity and protein kinase function that activates NRF2 via p62 phosphorylation, increases nucleotide synthesis via PRPS1 phosphorylation, and promotes EMT via indirect E-cadherin repression. This isoform switch is driven in part by MYC-dependent induction of hnRNPH1 and hnRNPH2. (**C**). *GLS1* mutually exclusive alternative splicing of exon 15 vs. exons 16–19 generates GAC and KGA isoforms, respectively. GAC is a shorter, highly active isoform enriched in cancer that promotes glutamine utilization, TCA cycle flux, and biosynthesis. KGA is a longer, lower-activity isoform associated with differentiated tissues. Isoform selection is regulated by CFIm-mediated alternative polyadenylation, CCAT2-enhanced proximal site usage, and HuR-dependent promotion of KGA splicing and transcript stability. Figures were generated with BioRender.com.

Pyruvate kinase (PK), encoded by the paralogous genes *PKM* (pyruvate kinase muscle) and *PKLR* (pyruvate kinase liver/red blood cell), catalyzes the final, rate-limiting step of glycolysis, transferring a phosphate from phosphoenolpyruvate (PEP) to ADP to generate pyruvate and ATP [67]. *PKM* is the predominant pyruvate kinase gene expressed in proliferating cells and most cancers, whereas *PKLR* expression is largely restricted to differentiated tissues such as liver and erythrocytes [68]. The pre-mRNA of *PKM* undergoes mutually exclusive alternative splicing to generate either the PKM1 or PKM2 isoform (Figure 1A), which differ in enzymatic activity and regulatory properties [69]. This splicing decision is governed by selective inclusion of exon 9 (PKM1) or exon 10 (PKM2) and is regulated by a coordinated network of splicing factors acting on cis-regulatory elements [70].

In cancer cells, hnRNPA1, hnRNPA2, and PTB bind ISS elements flanking exon 9 to repress its inclusion, thereby favoring exon 10 selection and *PKM2* expression [71]. Expression of these repressors is further driven by oncogenic transcription factors such as MYC, which links oncogenic signaling pathways to isoform selection [72]. In contrast, SRSF3 promotes exon 10 inclusion by binding to an ESE in exon 10, which promotes *PKM2* expression even in the presence of competing repressors [73]. Notably, SRSF3 is frequently upregulated in cancer and has been linked to MYC-driven transcriptional programs [74]. In addition, LINC01852-mediated regulation of SRSF5 [75,76], hnRNPC-dependent m6A-linked regulation [77], and lactate-induced hnRNPA1 K350 lactylation [78] bias *PKM* splicing toward *PKM2* in specific tumor contexts.

PKM1 functions as a constitutively active tetramer and is expressed in differentiated tissues, whereas PKM2 exists in a dynamically regulated equilibrium between a low-activity dimer and a high-activity tetramer [79]. The glycolytic intermediate fructose-1,6-bisphosphate (FBP) allosterically promotes PKM2 tetramerization in highly glycolytic cancer cells [79]. However, this effect is counteracted by oncogenic signaling pathways, as phosphotyrosine-containing peptides bind PKM2 and displace FBP, thereby destabilizing the tetramer and shifting PKM2 toward a low-activity state [80,81]. This reduced activity promotes the accumulation of upstream glycolytic intermediates that can be diverted into biosynthetic pathways while maintaining lactate production characteristic of the Warburg effect [68].

The dimeric form of PKM2 also has non-metabolic activity, functioning as a transcriptional co-activator through direct interactions with β-catenin and HIF-1α and, unexpectedly, as a protein kinase for STAT3 (Tyr705) and histone H3 (Thr11) [82,83,84,85,86]. However, the relative contribution of these metabolic and non-metabolic functions is context-dependent across cancer types. Importantly, regardless of the underlying mechanism, suppression of PKM2 is consistently associated with decreased tumor growth [87]. Collectively, these findings establish *PKM* splicing as a paradigm through which alternative splicing can reprogram metabolic flux in cancer, while also revealing isoform-specific vulnerabilities that may be exploited therapeutically.

### 5.2. Ketohexokinase

Ketohexokinase (KHK) phosphorylates fructose to form fructose-1-phosphate (F1P), enabling fructose-derived carbon to enter glycolysis downstream of phosphofructokinase-1 (PFK-1) [88]. Bypassing this regulatory step leads to an accumulation of glycolytic intermediates that can be diverted into de novo lipogenesis and triglyceride synthesis [89]. This metabolic routing has been strongly linked to metabolic dysfunction-associated steatotic liver disease (MASLD) and its progressive form, metabolic dysfunction-associated steatohepatitis (MASH), both of which are associated with an increased risk of gastrointestinal malignancies [90]. Interestingly, fructose metabolism is not essential for tumor growth, suggesting that the contribution of KHK to cancer is likely context dependent and may extend beyond its canonical role in fructose catabolism [91].

The *KHK* gene undergoes mutually exclusive alternative splicing to generate KHK-A and KHK-C [88] (Figure 1B). This splicing event involves the selection of alternative exons 3A and 3C within the catalytic domain, producing isoforms with distinct catalytic properties [88]. KHK-C is the predominant isoform in normal liver and exhibits high catalytic activity toward fructose, whereas KHK-A displays significantly reduced enzymatic activity and is more broadly expressed across tissues [92,93]. In hepatocellular carcinoma (HCC), there is a well-characterized isoform switch from KHK-C to KHK-A, which is driven in part by MYC-induced upregulation of hnRNPH1 and hnRNPH2 [94].

The KHK-A isoform has non-canonical functions independent of its metabolic activity. For example, KHK-A can function as a protein kinase, phosphorylating substrates such as PRPS1 to promote nucleotide biosynthesis and support proliferative capacity [94]. KHK-A also phosphorylates p62 to activate NRF2 and promote epithelial–mesenchymal transition through indirect repression of E-cadherin [95,96]. Furthermore, genetic knockdown or pharmacological inhibition of KHK-A reduces tumor growth, invasion, and metastasis across multiple cancer models [95,96,97,98]. Overall, these findings support the therapeutic potential of splice-modulating approaches to selectively target KHK-A.

### 5.3. GLS1 Splice Variants GAC/KGA

Many tumors depend on glutamine to replenish TCA cycle intermediates, sustain nucleotide and amino acid synthesis, and maintain cellular antioxidant capacity [99,100,101]. At the core of this pathway, glutaminase (GLS) converts glutamine to glutamate and ammonia, thereby controlling the entry of glutamine-derived carbon into downstream metabolic pathways [102,103]. GLS is encoded by *GLS1* and *GLS2*, with *GLS1* serving as the predominant source of GLS activity in proliferating cells and many cancers, whereas *GLS2* expression is more commonly associated with differentiated tissues and p53-dependent metabolic regulation [104,105]. The *GLS1* pre-mRNA consists of 19 exons and undergoes alternative splicing coupled to alternative polyadenylation to generate two isoforms, glutaminase C (GAC) and kidney-type glutaminase (KGA) [106,107,108] (Figure 1C). Inclusion of exon 15 together with the usage of a proximal intronic polyadenylation site within intron 14 generates the truncated GAC isoform, which lacks exons 16–19 [106]. Alternatively, skipping exon 15 permits splicing to downstream exons 16–19, which produces the full-length KGA isoform [106]. The GAC isoform is preferentially enriched across multiple tumor types, including colorectal cancer, lung adenocarcinoma, renal cell carcinoma, and AML [109,110,111,112,113,114,115,116,117].

The GAC and KGA isoforms exhibit distinct biochemical and subcellular properties [118]. GAC contains a shorter, relatively disordered C-terminal region and displays higher catalytic efficiency, whereas KGA possesses a longer, more structured C-terminal domain with ankyrin-repeat-like features and lower basal activity [118]. Glutaminase activity is regulated through the interplay of oligomerization and ligand-dependent activation, with tetramerization enhancing substrate affinity and inorganic phosphate promoting catalytic turnover [118]. GAC exhibits a greater intrinsic propensity to adopt these active states, a property that may be linked to its distinct C-terminal structure, and is more responsive to inorganic phosphate than KGA, providing a mechanistic basis for its enhanced activity [118]. GAC is preferentially enriched in mitochondria whereas KGA is more diffusely distributed in the cell, despite both isoforms sharing an N-terminal mitochondrial targeting sequence [119]. These differences in subcellular localization may be mediated by their distinct C-terminal domains, which influence mitochondrial retention rather than import.

The mechanisms governing *GAC* versus *KGA* expression are not fully resolved and involve multiple layers of RNA processing. For example, the cleavage factor Im (CFIm) complex binds UGUA elements within intron 14 and promotes proximal polyadenylation, thereby favoring GAC production, whereas loss of CFIm shifts processing toward KGA [107]. This axis is further modulated by oncogenic noncoding RNAs such as *CCAT2*, which interacts with CFIm to enhance GAC expression [107]. Furthermore, the RNA-binding protein HuR (Human antigen R, also known as ELAV-like protein 1) promotes KGA splicing while simultaneously increasing overall *GLS1* mRNA stability and translation, demonstrating that isoform ratio and total enzyme abundance can be independently regulated [108]. Overall, these reports highlight that the multilayered regulation of GLS1 creates multiple therapeutic entry points, including targeting isoform selection, enzymatic activity, or the RNA processing pathways that favor GAC expression.

## 6. Metabolite-Dependent Regulation of Splicing

### 6.1. Methionine Metabolism and Splicing of MAT2A

*MAT2A* encodes methionine adenosyltransferase 2A, the enzyme responsible for synthesizing S-adenosylmethionine (SAM), which is the universal donor for methylation of proteins and nucleic acids [120]. Methylation of Sm proteins is required for assembly of snRNPs, linking SAM availability to splicing regulation [121].

**Figure 2:**
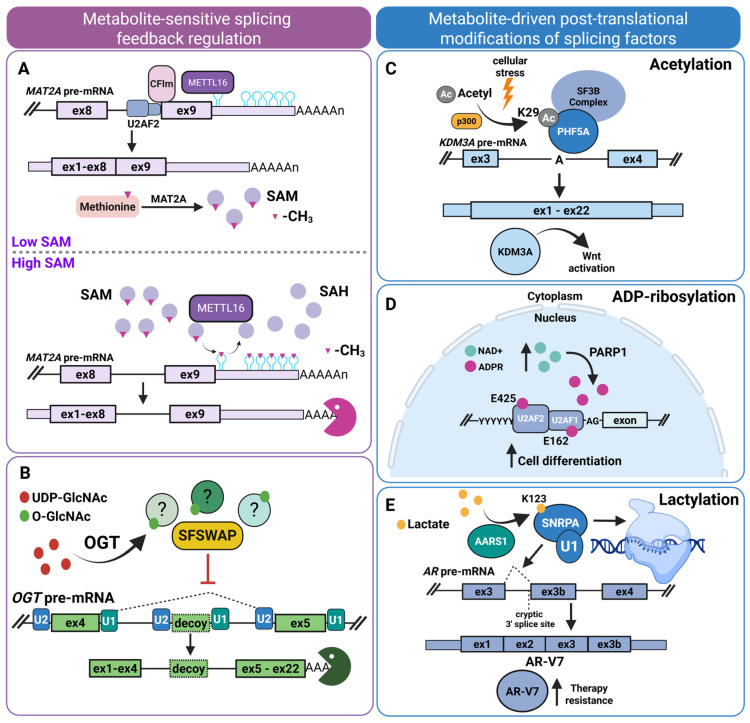
Metabolites regulate splicing through feedback circuits and post-translational modifications. (**A**). S-adenosylmethionine (SAM) regulates splicing of *MAT2A* pre-mRNA through the RNA methyltransferase METTL16. Under low SAM conditions, METTL16 remains bound to hairpin 1 in the *MAT2A* 3′UTR, which promotes CFIm-regulated splicing of the terminal intron, increasing *MAT2A* expression and restoring SAM levels. High SAM concentration drives METTL16 to methylate the 3′UTR hairpins of *MAT2A*. The high enzymatic activity of METTL16 reduces its dwell time on *MAT2A* 3′UTR, promoting intron retention and nuclear degradation of the *MAT2A* mRNA. (**B**). O-GlcNAcylation regulates intron retention of *OGT* pre-mRNA. Elevated UDP-GlcNAc levels lead to globally increased OGT-mediated O-GlcNAcylation of proteins that promote retention of intron 4 in the *OGT* mRNA, leading to nuclear sequestration of the transcript and reduced *OGT* expression. The splicing factor SFSWAP promotes *OGT* intron retention through modulation of spliceosome engagement at a decoy exon, which is presumed to be influenced by global O-GlcNAcylation of proteins, although these proteins have not yet been defined (designated with “?”). (**C**). Acetylation of splicing factors. Acetyl-CoA-dependent acetylation at K29 of PHF5A (also known as SF3B7, a component of the SF3B complex) by p300 under cellular stress stabilizes spliceosome assembly and suppresses aberrant splicing. This promotes proper splicing of *KDM3A* pre-mRNA, increasing expression of functional KDM3A and activating downstream oncogenic pathways such as Wnt signaling. (**D**). NAD+ drives ADP-ribosylation of U1AF. Increased nuclear NAD^+^ induces NAD^+^-dependent activation of PARP1 which drives ADP-ribosylation (ADPR) of U2AF components U2AF1 and U2AF2 at E425 and E162, respectively. This enhances their interaction with 3′ splice site elements, including the polypyrimidine tract and AG dinucleotide, which promotes splice-site recognition and contributes to context-specific splicing programs associated with cellular differentiation. (**E**). Protein Lactylation enhances splicing through SNRPA. Lactate accumulation in the tumor microenvironment induces lysine lactylation of the spliceosomal protein SNRPA, a component of the U1 snRNP. Lactylation enhances SNRPA association with chromatin and nascent pre-mRNA, promoting efficient co-transcriptional spliceosome engagement. This enhances exon definition and facilitates usage of nearby splice sites, including a cryptic 3′ splice site in the androgen receptor (*AR*) pre-mRNA, resulting in the production of an oncogenic isoform (AR-V7) that drives therapy resistance in prostate cancer.

SAM homeostasis is maintained through a feedback mechanism that regulates *MAT2A* expression via splicing of its terminal intron [122] (Figure 2A). Under conditions of low intracellular SAM, the m6A RNA methyltransferase METTL16 remains stably bound to a conserved hairpin (hp1) in the *MAT2A* 3′ UTR and promotes splicing of the terminal retained intron, stabilizing *MAT2A* mRNA and increasing production of MAT2A protein to restore SAM levels [122]. This process requires CFIm, which is proposed to facilitate spliceosome assembly at a weak PPT, potentially through enhanced recruitment of U2AF [122,123,124,125]. However, the precise mechanism remains incompletely defined. When SAM levels are elevated, increased METTL16 catalytic activity decreases its dwell time at hp1, thereby limiting its ability to promote splicing [125]. This results in retention of the terminal intron and nuclear degradation of the transcript, reducing *MAT2A* expression. This regulatory circuit provides a direct example where an enzyme product feeds back to regulate enzyme expression through its impact on splicing.

The SAM-dependent methyltransferases protein arginine methyltransferase 5 (PRMT5) supports proliferation and survival in glioblastoma, mantle cell lymphoma, AML, pancreatic cancer, and non-small-cell lung cancer [126,127]. Inhibition of PRMT5 causes widespread splicing defects, including intron retention and exon skipping in genes required for tumor growth. For example, loss of MTAP (methylthioadenosine phosphorylase), which is frequently co-deleted with the tumor suppressor CDKN2A on chromosome 9p21, leads to accumulation of methylthioadenosine (MTA), a byproduct of polyamine metabolism that inhibits PRMT5. Consequently, MTAP-deficient tumors (e.g., glioblastoma, pancreatic ductal adenocarcinoma, mesothelioma, and lung adenocarcinoma) with reduced PRMT5 activity become hypersensitive to pharmacologic inhibition of PRMT5 [128]. PRMT5 inhibitors, including GSK3326595 (pemrametostat), JNJ-64619178, and PF-06939999, are currently being evaluated in clinical trials to exploit this metabolic vulnerability [129].

SAM is also a precursor for the generation of polyamines through SAM decarboxylase. Polyamines such as spermine and spermidine are naturally occurring cationic small molecules that bind to negatively charged sites in macromolecules to regulate diverse cellular functions [130]. Polyamines bind to acidic motifs in the U2 snRNP-associated SF3 complex and reduce their phosphorylation through a mechanism referred to as metabolic shielding [131]. Inhibition of de novo polyamine synthesis therefore increases phosphorylation of spliceosomal proteins in the SF3 complex and alters splicing broadly. These recent findings may explain earlier work showing that polyamines suppress inclusion of a poison exon in the mRNA encoding spermidine/spermine N1-acetyltransferase (SSAT), the rate-controlling enzyme in the interconversion of spermidine and spermine [132]. Inclusion of the poison exon in turn causes degradation of the *SSAT* mRNA through nonsense-mediated decay.

### 6.2. O-GlcNAc Influences Splicing

The addition of O-linked N-acetylglucosamine (O-GlcNAc) to proteins by O-GlcNAc transferase (OGT) is a reversible post-translational modification that influences a variety of cellular processes including RNA processing [133,134]. Elevated O-GlcNAc levels are associated with increased cell proliferation and tumor progression [135]. O-GlcNAc homeostasis is maintained, in part, through an O-GlcNAc-dependent feedback mechanism that regulates splicing of a retained intron in the *OGT* transcript [57] (Figure 2B). In this context, transcripts containing retained introns are sequestered in the nucleus, thereby preventing translation [57].

Splicing of intron 4 of the *OGT* pre-mRNA is responsive to UDP-GlcNAc, which is the substrate for OGT and is produced via the hexosamine biosynthetic pathway [133]. This pathway integrates multiple nutrient inputs including glucose, glutamine, acetyl-CoA, and nucleotide metabolism [133]. When UDP-GlcNAc levels are elevated, intron 4 of *OGT* is retained, resulting in nuclear sequestration of the transcript and decreased *OGT* expression [57]. In contrast, when UDP-GlcNAc levels are reduced, intron 4 is spliced out allowing generation of mature *OGT* mRNA [57].

Recent work has begun to identify the molecular regulators of this process. A CRISPR-based screen with an O-GlcNAc reporter system identified the splicing factor SFSWAP as a key mediator of *OGT* intron 4 retention [136]. Loss of SFSWAP via siRNA knockdown significantly reduced intron 4 retention, further indicating that SFSWAP promotes intron retention [136]. Mechanistically, SFSWAP appears to influence spliceosome engagement at a decoy exon within intron 4, which weakens spliceosome assembly at the canonical splice sites, thereby promoting intron retention [136]. SFSWAP becomes highly phosphorylated following OGT inhibition, raising the possibility that SFSWAP functions downstream of a metabolite-sensitive signaling pathway that modulates spliceosome activity. Given that pharmacologic inhibition of OGT also suppresses proliferation and induces apoptosis in breast, colon cancer, and prostate cancer models [137,138,139], strategies aimed at altering splicing of the *OGT* pre-mRNA may represent a novel therapeutic approach to treat various cancers.

Consistent with this concept, a recent study has identified small molecules that perturb the retained intron regulating expression of *OGT* and the O-GlcNAc hydrolase *OGA* [140]. High-throughput screening efforts yielded compounds such as GSK690693 and Y-33075 that broadly modulate splicing, leading to widespread intron retention or altered cassette exon inclusion, respectively. Moreover, these perturbations disrupt the retained intron-mediated feedback mechanism controlling O-GlcNAc homeostasis, lowering both OGT and OGA protein levels, which paradoxically increases global O-GlcNAcylation due to reduced OGA-mediated turnover [140]. These findings highlight how pharmacologic modulation of splicing can indirectly influence O-GlcNAc signaling pathways. However, it remains to be seen whether RNA-based approaches, such as splice-switching antisense oligonucleotides (ASOs), may provide a more precise strategy to manipulate OGT detained intron splicing and modulate O-GlcNAc homeostasis.

### 6.3. Acetyl-CoA and NAD+ Influence Splice Site Selection

Fluctuations in Acetyl-CoA and nicotinamide adenine dinucleotide (NAD+) in the tumor microenvironment (TME) modulate acetylation and ADP-ribosylation of the splicing machinery and influence splicing indirectly through modification of the underlying chromatin state. Acetyl-CoA generated from glucose, lipid, and amino acid metabolism is often reduced in cancer cells under nutrient stress [141]. Acetyl-CoA serves as a critical metabolic sensor that shuttles between cellular compartments, including the nucleus and mitochondria, to modulate acetylation of proteins.

For example, nutrient deprivation promotes p300-mediated acetylation of PHF5A (also known as SF3B7), a highly conserved scaffold component of the SF3B complex within the U2 snRNP [142] (Figure 2C). Acetylation of PHF5A^K29^ stabilizes the U2 snRNP complex and suppresses aberrant mRNA alternative splicing. Mechanistically, PHF5A^K29^ acetylation reduces intron 3 retention of *KDM3A*, which encodes a histone demethylase that removes H3K9 mono and dimethylation marks [142]. This intron contains a PTC that triggers nonsense-mediated decay. As a result, PHF5A acetylation promotes the production of functional *KDM3A* mRNA and protein. Similarly, the PHF5^K29Q^ mutation decreases intron 3 retention and enhances *KDM3A* expression, promoting Wnt signaling and colorectal tumorigenesis [143]. Together these findings illustrate how metabolic cues, via acetyl-CoA dependent protein acetylation, can influence splicing and downstream oncogenic pathways.

NAD+ also regulates splicing through epigenetic and post-translational mechanisms [144]. Cancer cells reprogram their metabolic landscape to sustain elevated NAD+ levels, primarily through the Warburg effect and the NAMPT (nicotinamide phosphoribosyltransferase)-driven salvage pathway, which in turn fuels NAD+-dependent enzymes such as PARP1 (poly(ADP-ribose) polymerase 1). PARP1 directly modulates the splicing machinery through site-specific ADP-ribosylation (ADPRylation) [145] of core spliceosomal components, including U2AF1 and U2AF2 (Figure 2D), thereby enhancing their recognition of 3′ splice sites and RNA-binding affinity to drive context-specific splicing isoforms. This mechanism links NAD+ metabolism to alternative splicing programs that support oncogenic phenotypes and enable adaptation to metabolic stress.

### 6.4. Lactate Alters Splicing in Cancer

Lactate produced as a byproduct of increased tumor glycolysis modulates the tumor landscape through multiple functions by acting as a primary fuel source, a signaling molecule, and a context-specific regulator of the transcriptome [146]. Lactate is generated from pyruvate by the redox enzyme lactate dehydrogenase (LDH) in the cytosol, but recent evidence also highlights a mitochondrial venting mechanism where pyruvate in the mitochondrial matrix is converted to lactate and exported via the mitochondrial pyruvate carrier (MPC) to maintain redox balance and limit oxidative stress [147]. Furthermore, lactate acts as a non-metabolic mitochondrial messenger that directly stimulates the electron transport chain, prompting a shift from glycolysis to oxidative phosphorylation to optimize energy utilization in nutrient-poor environments [148].

Excessive accumulation of lactate in the TME, derived from tumor cell glycolysis, can be sensed by tumor-infiltrating immune cells such as regulatory T cells (Tregs). Lactate uptake by Tregs specifically in the TME triggers USP39-mediated CTLA-4 pre-mRNA splicing to facilitate CTLA-4 expression in a Foxp3-dependent manner, contributing to the efficacy of CTLA-4 blockade in antitumor immunity [149]. In addition, lactate bridges metabolism and gene expression through lysine lactylation (Kla), a post-translational modification that uses lactate-derived lactyl-CoA as a substrate [150,151]. In prostate cancer, lactate derived from cancer-associated fibroblasts (CAFs) induces the lactylation of the spliceosome component SNRPA at K123 (Figure 2E), which enhances its chromatin binding and promotes alternative splicing towards the AR-v7 splice variant. AR-v7 is constitutively active, which confers resistance to androgen deprivation therapy [152]. Other examples include Nucleolin (NCL) lactylation, which alters MADD splicing to generate an isoform that activates mitogen activated kinase (MAPK) signaling in intrahepatic cholangiocarcinoma [153]. In bladder cancer, enhanced lactylation of hnRNPA1 facilitates *PKM* pre-mRNA splicing from the PKM1 to the pro-glycolytic PKM2 isoform, establishing a self-sustaining feedforward loop that accelerates glycolytic flux [78], while hnRNPC lactylation alters *PAK6* splicing to drive pancreatic cancer metastasis [154]. Collectively, these findings identify lactate as a metabolic regulator that induces site specific lactylation of RNA binding proteins in the splicing machinery, selectively reshaping the proteome to favor oncogenic adaptation and therapeutic evasion.

## 7. Metabolic Stress

### 7.1. Hypoxia-Driven Changes in Splicing

Hypoxia is a defining feature of the TME and imposes a fundamental constraint on cellular metabolism [155]. At the molecular level, reduced oxygen tension stabilizes the transcription factor hypoxia inducible factor 1α (HIF-1α) [156]. Under normoxic conditions, HIF-1α is hydroxylated by oxygen-dependent prolyl hydroxylase domain (PHD) enzymes, creating a binding site for the von Hippel–Lindau (VHL) E3 ubiquitin ligase complex and targeting HIF-1α for proteasomal degradation [157]. As oxygen levels decline, impaired PHD activity leads to stabilized HIF-1α, enabling its nuclear accumulation, dimerization with ARNT (HIF-1β), and activation of transcriptional programs that promote adaptation to hypoxia [155]. These programs include induction of angiogenesis, suppression of mitochondrial oxidative phosphorylation, modulation of redox homeostasis, altered glycolytic flux, and attenuation of apoptosis [155]. Hypoxia is also associated with widespread alterations in splicing that are thought to reinforce these processes [158,159]. However, the mechanisms underlying these changes have only recently begun to be elucidated.

Hypoxic signaling can directly influence splice-site selection. Transcriptome-wide analyses of hepatocellular carcinoma cells revealed that hypoxia induces widespread changes in alternative splicing, affecting genes involved in metabolism, stress adaptation, and cellular survival [160]. HIF target genes preferentially exhibited exon inclusion, whereas genes repressed under hypoxic conditions more commonly underwent exon skipping. HIF activity, not hypoxia per se, was both necessary and sufficient to increase exon inclusion of several HIF target genes. For example, HIF activity was sufficient to promote inclusion of exon 4 in pyruvate dehydrogenase kinase 1 (*PDK1*), whereas transcriptional activation through non-HIF-responsive promoters reduced exon inclusion. Replacement of the HIF-1α transactivation domain with either VP16 or E2F1 transactivation domains did not alter the resulting splicing pattern, indicating that splice-site selection does not require a unique biochemical property of HIF-1α [160]. Instead, transcriptional activation of HIF-responsive genes was sufficient to influence splicing outcomes, supporting a model in which HIF-dependent alternative splicing arises through co-transcriptional coupling between promoter activation, RNA pol II activity, and spliceosome assembly.

Hypoxic signaling also influences alternative splicing indirectly through regulation of the splicing machinery itself. In prostate cancer cells, hypoxia induces alternative splicing of genes involved in proliferation, migration, adhesion, and metabolism, while simultaneously increasing expression of genes encoding splicing regulators [161]. Hypoxia also increases expression of the genes encoding the splicing factor kinases CLK1 and CLK3 (cdk like kinases). The *CLK1* and *CLK3* promoters contain hypoxia response elements and are regulated by HIF signaling linking hypoxic signaling to altered splice-site selection. Depletion of *CLK1* alters splicing of hypoxia-responsive transcripts, supporting a functional role for CLK-mediated regulation of alternative splicing under hypoxic conditions [161]. These findings indicate that HIF can influence alternative splicing through co-transcriptional regulation of target genes and through transcriptional control of core splicing regulators.

Nuclear organization provides an additional layer of hypoxia-responsive splicing regulation. The long non-coding RNA metastasis-associated lung adenocarcinoma transcript 1 (MALAT1), a hypoxia-inducible transcript enriched at nuclear speckles, regulates a large proportion of hypoxia-responsive splicing events across multiple cancer cell lines [162]. MALAT1 interacts with SRSF1 and promotes formation of SRSF1-containing condensates, thereby facilitating recruitment of splicing machinery to hypoxia-responsive transcripts. MALAT1 also enhances the specificity of SRSF1 binding and promotes interactions between SRSF1 and elongating RNA pol II, supporting efficient co-transcriptional splicing [162]. Loss of either MALAT1 or SRSF1 impairs hypoxia-responsive splicing and reduces expression of hypoxia-responsive genes [162]. Together, these findings suggest that hypoxia-responsive splicing is regulated through transcriptional programs, splicing factor abundance, and through the spatial organization of RNA processing within the nucleus.

**Figure 3:**
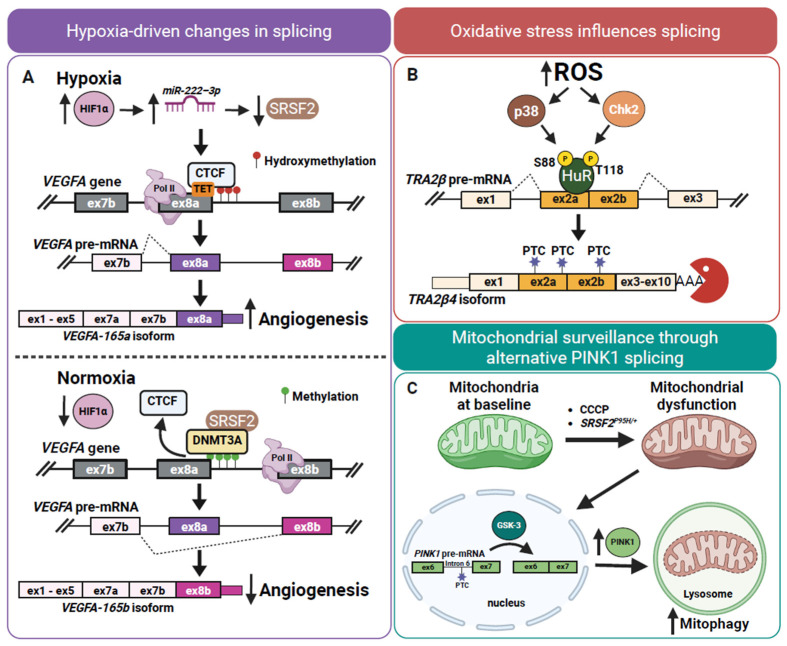
Metabolic stress alters splicing. (**A**). Hypoxia-driven alternative splicing of *VEGFA*. Stabilization of HIF-1α induces *miR-222-3p* expression, which suppresses *SRSF2* expression and alters epigenetic regulation at the *VEGFA* locus. Under hypoxia, reduced DNMT3A-mediated methylation and increased hydroxymethylation promote CTCF binding and RNA pol II pausing at exon 8a, favoring inclusion of exon 8a and production of the pro-angiogenic *VEGF-A165*a isoform. Under normoxic conditions, *SRSF2* expression is increased, resulting in an SRSF2-DNMT3A coordinated methylation of *VEGFA* that favors exon 8b inclusion, generating the anti-angiogenic *VEGF-A*165b isoform. (**B**). Oxidative stress alters splicing factor activity. Reactive oxygen species (ROS) activate p38 MAPK and Chk2, which phosphorylate HuR at S88 and T118, enhancing its binding to *TRA2β* pre-mRNA. This promotes inclusion of exon 2, resulting in the *TRA2β4* isoform, which contains multiple PTCs. TRA2β regulates numerous downstream splicing targets, thereby linking redox signaling to broader splicing programs. (**C**). Mitochondrial surveillance through alternative splicing of *PINK1*. Mitochondrial dysfunction (caused by CCCP or the oncogenic splicing factor mutation *SRSF2^P95H/+^*) promotes splicing of *PINK1* pre-mRNA to remove intron 6, stabilizing *PINK1* mRNA and increasing mitophagy. In contrast, intron 6 retention introduces a PTC and targets the transcript for degradation. This process is regulated in part by GSK-3 and links mitochondrial stress to spliceosome activity.

The diverse mechanisms linking hypoxic signaling to splice-site selection are perhaps best illustrated by vascular endothelial growth factor A (VEGFA), one of the most extensively studied hypoxia-responsive alternatively spliced transcripts. HIF-1α promotes angiogenesis by increasing expression of vascular endothelial growth factor A (VEGFA), which exists as multiple isoforms broadly divided into pro- and anti-angiogenic families [163,164,165,166] (Figure 3A). These isoforms arise from alternative 3′ splice-site selection within terminal exon 8 (exon 8a vs. 8b), generating VEGF-Axxxa and VEGF-Axxxb isoforms (where xxx denotes protein length) that differ in their terminal six amino acids and mediate strong versus weak VEGFR2 activation, respectively. VEGF-A165a is the predominant isoform in most cancers [167,168,169], and its splicing is regulated by SR proteins including SRSF1, SRSF6, and SRSF2 [170,171,172]. Recent work implicates a HIF-1α–miR-222-3p–SRSF2 axis that shifts splicing toward VEGF-A165a [173,174]. Loss of SRSF2 is associated with altered epigenetic regulation at exon 8, including reduced DNMT3A-associated methylation, increased hydroxymethylation, CTCF binding, and RNA Pol II pausing, collectively favoring exon 8a inclusion [173]. However, these findings are largely limited to in vitro breast cancer models. MALAT1 and SRSF1 also regulate VEGFA isoform expression, linking nuclear speckle-associated RNA processing to angiogenic signaling [175]. Additional splicing factors have also been implicated in hypoxia-associated splicing of *VEGFA*, though the underlying mechanisms remain incompletely defined [176,177].

Hypoxia is also associated with alternative splicing of *BNIP3*, *BNIP3L*, and *TCF3* [178,179,180], although the mechanisms have only been defined in a limited number of cancer models. Together, these observations demonstrate that hypoxic signaling can influence splice-site selection through multiple layers of regulation, ranging from co-transcriptional splicing and splicing factor expression to nuclear speckle-associated RNA processing. However, the relative contribution of these pathways across different tumor types and transcript targets remains unclear.

### 7.2. Oxidative Stress Influences Splicing

Oxidative stress in the TME arises from processes such as reactive oxygen species (ROS) production, mitochondrial electron transport chain dysfunction, altered NAD(P)H/NAD(P)+ balance, and lipid peroxidation [181]. In cancer cells, these redox perturbations can reshape gene expression programs [181]. Oxidative stress can also alter splicing through direct modulation of splicing factor activity or through changes in transcriptional and chromatin states that influence splice-site recognition [182]. However, the downstream functional consequences of many of these splicing alterations remain incompletely defined and warrant further investigation.

For example, in human colorectal cancer cells, oxidative stress-induced activation of p38 MAPK and checkpoint kinase 2 (Chk2) promotes phosphorylation of HuR at residues S88 and T118 (Figure 3B), altering its RNA-binding activity and enabling association with exon 2a of *TRA2β* pre-mRNA [183]. This interaction promotes inclusion of exon 2, which introduces a PTC and generates the nonproductive TRA2β4 splice isoform [183]. As TRA2β is a general splicing regulator, stress-dependent modulation of *TRA2β* splicing provides a mechanism for amplifying oxidative signals into broader alternative splicing networks that may influence cancer cell proliferation, survival, and therapeutic resistance [184,185].

Oxidative stress can also influence splicing through chromatin-dependent mechanisms that act co-transcriptionally. A notable example is the Brahma-BRCA1-CstF regulatory axis, in which oxidative stress reduces levels of the SWI/SNF chromatin remodeler Brahma (BRM) in human cancer cell lines, a factor that normally contributes to transcriptional regulation and chromatin accessibility [186]. Under normal conditions, BRM interacts with the BRCA1/BARD1 complex, which ubiquitinates the 50 kDa subunit of the 3′ end processing factor CstF, thereby inhibiting cleavage at proximal polyadenylation sites and promoting inclusion of distal terminal exons. Loss of BRM under oxidative stress disrupts this regulatory pathway, relieving inhibition of CstF and promoting cleavage at proximal polyadenylation sites [186]. As a result, oxidative stress induces shifts in alternative last exon usage and transcript shortening across a subset of genes, although the broader gene networks and functional consequences of these changes remain to be fully defined [186].

While these studies illustrate how oxidative stress can directly reshape splice-site selection, alternative splicing can also reciprocally regulate cellular responses to redox perturbations. In lung cancer, the tumor suppressor menin (MEN1) regulates ferroptotic sensitivity through co-transcriptional regulation of *CD44* splicing [187]. CD44 is a transmembrane adhesion receptor that undergoes extensive alternative splicing to generate a standard isoform (CD44s), which lacks variable exons 6–14, and multiple isoforms (CD44v) that include various combinations of these exons, each with distinct functional roles in cancer progression [188]. In particular, CD44 variant isoforms containing exons v8–10 promote tumor cell survival and stress adaptation by interacting with the cystine/glutamate antiporter SLC7A11 at the plasma membrane [189]. This interaction enhances cystine uptake and glutathione synthesis, thereby increasing resistance to oxidative stress and ferroptosis [189,190]. Mechanistically, menin interacts with the PAF1 complex to slow Pol II elongation of *CD44* pre-mRNA, facilitating the recruitment of splicing regulators such as ESRP1 and hnRNPLL [187]. This promotes exclusion of *CD44* variable exons and favors expression of the CD44s isoform [187]. In contrast, loss of menin increases Pol II elongation rates, reduces recruitment of splicing factors, and shifts splicing toward CD44v expression, which is associated with enhanced resistance to ferroptotic stress [187].

### 7.3. Mitochondrial Quality Control via Alternative Splicing

Mitochondrial stress is closely intertwined with these redox pathways, as mitochondria are a major source of intracellular ROS and play central roles in apoptosis and metabolite production. However, compared with oxidative stress-induced signaling, direct links between mitochondrial dysfunction and alternative splicing in cancer remain less well defined.

Recent work has identified a mechanism for mitochondrial surveillance that links mitochondrial dysfunction to a shift in splicing of the mitophagy regulator *PINK1* and reciprocally links altered splicing to mitochondrial dysfunction (Figure 3C). For example, *SRSF2^P95H/+^*, a common driver mutation in CMML, MDS, and AML, causes widespread changes in the splicing of nuclear encoded mitochondrial genes, impairs oxidative phosphorylation, and increases autophagic turnover of mitochondria (mitophagy) [191]. *SRSF2^P95H/+^* cells depend on this increased level of mitophagy for survival, as inhibition of mitophagy is lethal to cells with mutant SRSF2. Expression of mitophagy markers such as *OPTN* and *TOMM7* is elevated in cells from AML patients with *SRSF2^P95H/+^* compared to those with wild-type SRSF2, and in hematopoietic cell lines with this mutation [191]. Expression of the mitophagy activator PINK1 is also increased by introduction of the *SRSF2^P95H^* mutation, which arises through alternative splicing of *PINK1*. Two isoforms of *PINK1* mRNA can be detected in human cells, a fully spliced isoform and an isoform that retains intron 6. As intron 6 contains an in-frame PTC, retention of the intron activates nonsense-mediated decay. Therefore, interventions that promote intron 6 retention reduce overall levels of PINK1. In contrast, mitochondrial dysfunction caused by the *SRSF2^P95H/+^* mutation promotes removal of intron 6, stabilizing *PINK1* mRNA and increasing PINK1 protein levels and mitophagy. This splicing event is essential for the survival of *SRSF2^P95H/+^* cells, as interference with this splicing step reduces *PINK1* expression and mitophagy, resulting in selective lethality.

These observations implicate increased mitophagy as a disease marker and a therapeutic vulnerability in *SRSF2^P95H^* mutant MDS and AML. However, disruption of mitochondrial function also increases *PINK1* splicing in cells with wild-type *SRSF2.* For example, the protonophore CCCP, which reduces the mitochondrial membrane potential, also enhances *PINK1* splicing. This observation in cells with wild-type splicing factors suggests a general mechanism for mitochondrial surveillance in which mitochondrial dysfunction signals to the spliceosome to increase *PINK1* splicing in support of an increased demand for mitophagy [191]. In addition, the observation that mitochondrial dysfunction promotes *PINK1* splicing raises the intriguing possibility that other splicing changes observed in *SRSF2* mutant cells arise indirectly through disruption of mitochondrial function rather than a direct effect of mutated SRSF2 on splicing.

*PINK1* splicing is facilitated by GSK-3 [191], a CMGC kinase that phosphorylates multiple splicing factors, including SF3B1 and SRSF2 [53,54,55,192,193]. GSK-3 knockout or chemical inhibition alters splicing on a genome-wide scale in diverse cell types, including embryonic stem cells, cardiomyocytes, lymphocytes, and neurons. In hematopoietic cells, inhibition of GSK-3 alters the splicing of approximately 700 genes and promotes retention of intron 6 in *PINK1*, which reduces *PINK1* abundance, impairs mitophagy, and is therefore selectively lethal to *SRSF2^P95H/+^* hematopoietic cells [191]. How GSK-3 phosphorylation of splicing factors alters splicing of pre-mRNAs such as *PINK1* remains unclear at a molecular level, but these findings nevertheless suggest a potential therapeutic approach for splicing factor-mutant hematopoietic neoplasms.

## 8. Conclusions

The convergence of splicing and cellular metabolism represents a fundamental axis of tumor adaptation. Splicing is an essential function in gene expression, but, while homozygous loss of function in core splicing factors is lethal, heterozygous missense mutations in core splicing factors are drivers of hematologic and solid malignancies, and in some cases these mutations significantly alter cellular metabolism. Alternative splicing is also a prevalent downstream consequence of oncogenic signaling that can regulate metabolic states by generating enzymatic isoforms with distinct catalytic, regulatory, and non-canonical functions. Conversely, metabolic cues such as nutrient availability, redox state, and metabolite flux feed back to influence splicing. This bidirectional relationship enables cancer cells to rapidly and reversibly reprogram gene expression in response to environmental stress, which provides a selective advantage during tumor progression.

The reciprocal interplay between splicing and metabolism in cancer creates mechanistically distinct therapeutic opportunities. For example, ASOs that bind to pre-mRNAs offer a targeted approach to redirect specific isoform outcomes, including those of key metabolic enzymes such as PKM [194,195]. These studies establish a proof-of-principle that splice-switching strategies can be used to reprogram tumor metabolism and suggest that extending this approach to other enzymatic isoforms may uncover additional therapeutic vulnerabilities [196,197,198]. Synthetic lethal strategies exploiting combined vulnerabilities in splicing regulation and metabolic pathways may also provide enhanced therapeutic specificity [13,199,200,201,202].

Looking forward, continued advances in integrative “multi-omic” technologies may help to resolve the complexity of the splicing–metabolism interface. Long-read RNA sequencing is improving our ability to accurately quantify full-length isoforms, while metabolomics and isotope tracing provide functional insight into pathway utilization. When combined with proteomics and emerging approaches such as single-cell and spatial transcriptomics, these tools offer the potential to define context-specific splicing programs and their metabolic consequences across tumor types and microenvironmental niches [203,204,205,206,207,208,209].

Despite substantial progress, the mechanistic links between metabolic signaling and splicing remain incompletely defined. A deeper understanding of how specific metabolites, post-translational modifications, and chromatin states influence spliceosome dynamics will be critical for identifying actionable vulnerabilities. Moreover, the context dependence of many splicing events underscores the need to define tumor subtype-specific dependencies and to develop biomarkers that can stratify patients based on splicing or metabolic phenotypes.

## Data Availability

Not applicable.
